# Formation of Non-Nucleoplasmic Proteasome Foci during the Late Stage of Hyperosmotic Stress

**DOI:** 10.3390/cells10092493

**Published:** 2021-09-21

**Authors:** Jeeyoung Lee, Ly Thi Huong Luu Le, Eunkyoung Kim, Min Jae Lee

**Affiliations:** 1 Department of Biochemistry and Molecular Biology, Seoul National University College of Medicine, Seoul 03080, Korea; onnu2@snu.ac.kr (J.L.); huongluuly94@snu.ac.kr (L.T.H.L.L.); eunk.kek@snu.ac.kr (E.K.); 2 Department of Biomedical Sciences, Seoul National University Graduate School, Seoul 03080, Korea

**Keywords:** proteasome, hyperosmotic stress, nuclear foci, insulator body, nuclear pore complex, phase separation, liquid droplet, nucleocytoplasmic transport, stress granule

## Abstract

Cellular stress induces the formation of membraneless protein condensates in both the nucleus and cytoplasm. The nucleocytoplasmic transport of proteins mainly occurs through nuclear pore complexes (NPCs), whose efficiency is affected by various stress conditions. Here, we report that hyperosmotic stress compartmentalizes nuclear 26S proteasomes into dense nuclear foci, independent of signaling cascades. Most of the proteasome foci were detected between the condensed chromatin mass and inner nuclear membrane. The proteasome-positive puncta were not colocalized with other types of nuclear bodies and were reversibly dispersed when cells were returned to the isotonic medium. The structural integrity of 26S proteasomes in the nucleus was slightly affected under the hyperosmotic condition. We also found that these insulator-body-like proteasome foci were possibly formed through disrupted nucleus-to-cytosol transport, which was mediated by the sequestration of NPC components into osmostress-responding stress granules. These data suggest that phase separation in both the nucleus and cytosol may be a major cell survival mechanism during hyperosmotic stress conditions.

## 1. Introduction

Cells have multiple systems to maintain proteome integrity in response to various types of environmental stress. Misfolded proteins arising from stress-induced denaturation, mutation, and posttranslational modifications are cleared preferentially by the ubiquitin-proteasome system [1,2,3]. Reversible compartmentalization of potentially toxic proteins into membraneless organelles such as nuclear stress bodies and cytoplasmic stress granules is one of the pro-survival mechanisms of cells under substantial cellular stress [4]. Many of these adaptation strategies seem to have been evolutionarily conserved from yeast to mammals. While diverse nucleoplasmic and cytoplasmic protein inclusion bodies with distinct characteristics have been identified thus far [5,6], their biochemical and pathophysiological features remain largely uncharacterized. Liquid-liquid phase separation (LLPS) and promiscuous interactions among intrinsically disordered proteins are considered to be the main driving force behind the formation of stress-induced membraneless organelles including stress granules [7,8].

Stress granules are a type of ribonucleoprotein granules, where untranslated mRNA as well as RNA-binding and non-RNA-binding proteins are condensated [9]. In mammals, stress granules generated under heat, oxidative stress, osmotic stress, or proteasome inhibition conditions exist as soluble, liquid-like organelles [7]. This process is highly dynamic and reversible, and once these stresses are relieved, stress granules readily disperse into the cytosol. However, when the stress persists, excess amounts of mistranslated (and subsequently misfolded) proteins are produced and function as a key scaffold, which allows the components of stress granules to coalesce and generate less soluble granules, amorphous aggregates, and eventually amyloid deposits implicated in diverse neurodegenerative diseases [10].

Cells under hyperosmotic conditions experience free water efflux, cell shrinkage, and higher concentrations of their biomolecular contents. In addition to these mechanical consequences, many intracellular signaling pathways are profoundly altered for cell survival under such conditions. The mitogen-activated protein kinase (MAPK) p38 was reported to get activated under osmostress, which in turn phosphorylated proteasome subunits and inhibited proteasome activity, leading to global accumulation of polyubiquitylated proteasome substrates in the cell [11]. Studies have reported that more proteasomes are located in the nucleus (~1 µM) than in the cytosol (<200 nM) [12,13] and that their bidirectional transport, mainly through the nuclear pore complex, is tightly regulated depending on cellular status, such as proliferation, quiescence, and nutrient availability [14].

More recently, the Saeki group has identified that hyperosmorality induced the formation of proteasome-enriched nuclear foci via LLPS [15] and that these foci functioned as a nuclear proteolytic center for polyubiquitylated proteins and unassembled ribosomal proteins. Here, based on the results from the late stage of hyperosmotic stress using 200 mM NaCl, we report unique proteasome foci near the nuclear membrane, distinguished from the nucleoplasmic puncta observed by the Saeki group. The characteristics of these foci were similar to those of the previously identified chromatin insulator bodies [16]. These nuclear stress bodies were observed to form through LLPS and were unaffected by MAPK-mediated signaling cascades. When the cells were returned to the isotonic medium, the nuclear proteasome puncta rapidly dissolved. Proper nucleocytoplasmic transportation of nuclear proteasomes appeared to be critical for the foci formation. Collectively, these results suggest a plausible mechanism whereby the nuclear proteasome foci formation contributes to the survival of cells under osmotic stress conditions.

## 2. Materials and Methods

### 2.1. Plasmids, Antibodies and Reagents

Antibody sources and dilution factors used in this study were as follows: anti-β-actin (A1978, 1/10,000, Sigma, St. Louis, MO, USA), anti-PSMD1 (sc-514809, Santa Cruz Bio-technology, Dallas, TX, USA), anti-PSMC2 (sc-166972, 1/1000, Santa Cruz Biotechnology), anti-PSMA4 (PW8115, 1/5000, Enzo Life Science, Farmingdale, NY, USA), anti-GFP (2955S, 1/5000, Cell Signaling Technology, Danvers, MA, USA), anti-Flag (PA1-984B, 1/5000, Thermo Fisher Scientific, Waltham, MA, USA), anti-PABP (sc-32318, Santa Cruz Biotechnology, Dallas, TX, USA), anti-G3BP (ab56574, Abcam, Cambridge, UK), anti-Nup358 (ABN1385, MilliporeSigma, St. Louis, MO, USA), anti-Nup153 (ab96462, Abcam), anti-Nup107 (ab24609, Abcam), anti-Coilin (sc-56298, Santa Cruz Biotechnology), anti-PML (sc-966, Santa Cruz Biotechnology), MAPK family antibody sampler kit (9926T, Cell Signaling Technology), and phophorylated MAPK family antibody sampler kit (9910T, Cell Signal-ing Technology). The major biochemical reagents were as follows: NaCl, sucrose (Daejung Chemicals, Siheung, Korea); MNL-7423, SB202190, SP600125, and U0126 (Cayman Chemical, Ann Arbor, MI, USA), Importazole (Calbiochem), Leptomycin B (Cay-man); suc-LLVY-AMC (Bachem, Bubendorf, Switzerland); ATP (Apexbio, Houston, TX, USA); Coomassie Brilliant Blue R250. Fetal bovine serum (FBS) was purchased from Thermo Fisher Scientific. DMEM was purchased from WELGENE (Gyeongsan, Korea).

### 2.2. Cell Culture and Osmotic Stress

HCT116 (human colorectal carcinoma, ATCC #CCL-247) and HCT116-based knock-in cell lines (generated from the Saeki group [15]) were maintained in high-glucose DMEM (osmolality = 300 mOsmol) supplemented with 10% FBS, 1% penicillin/streptomycin, and 1% L-glutamine in a humidified environment containing 5% CO_2_ at 37 °C. Cells at 80% confluence were subjected to hyperosmotic shock by adding additional 200 mM NaCl at the indicated times. For the inhibitor experiments, cells were treated with 25 µM of inhibitor and 200 mM NaCl simultaneously. For washout experiments, the treated media were replaced with normal media before harvesting.

### 2.3. Western Blot Analysis and Subcellular Fractionation

Cultured cells were lysed in RIPA buffer (150 mM NaCl, 0.5 mM EGTA, 0.5% sodium deoxycholate, 1% SDS, 1% Triton X-100, 50 mM Tris-HCl (pH 7.4), protease inhibitor cocktail) and subsequently centrifuged at 16,000× *g* for 30 min at 4 °C to collect the supernatant. The pellets, as an insoluble fraction, were washed several times with RIPA buffer and then directly dissolved in SDS sample buffer. All samples were separated by SDS-PAGE and were subsequently transferred to a PVDF membrane. After blocking with 5% skim milk in TBST (10 mM Tris [pH 8.0], 150 mM NaCl, 0.5% Tween-20), membranes were incubated with primary antibodies at room temperature for 60 min. Membranes were then washed three times with TBST, incubated with secondary antibody for 60 min at room temperature, and washed three times with additional TBST before developing with enhanced chemiluminescence solution.

For nuclear fraction preparation, cells were collected in prechilled 1.5 mL tubes and dissolved in 300 µL of cytosolic extraction buffer (0.1% NP-40 in PBS) by pipetting. After incubation on ice for 5 min, the lysates were centrifuged for 5 min at 15,000× *g* at 4 °C to isolate the cytosolic fraction in the supernatant. The pellets were washed with PBS three times and resuspended in 100 µL RIPA buffer, vortexed for 30 s, and centrifuged for 10 min at 15,000× *g* at 4 °C. After centrifugation, the supernatants were collected as nuclear fractions.

### 2.4. Nondenaturing Gel Electrophoresis

NuPAGE 3–8% Tris-Acetate Protein Gels (Thermo Fisher Scientific) were used to separate the proteins in WCEs or purified proteasomes at 150 V for 4 h. Proteins in the gel were directly analyzed by in-gel suc-LLVY-AMC hydrolysis assay or transferred to PVDF membranes and then immunoblotted for proteasome subunits as described [17].

### 2.5. Immunofluorescence Microscopy and Fluorescence Recovery after Photobleaching (FRAP) Analysis

For immunofluorescence analysis, cultured cells on a cover glass were fixed with 4% paraformaldehyde in PBS for 15 min and then permeabilized with 0.5% (*v*/*v*) Triton X-100 in PBS. After blocking with 2% BSA in PBS, the cells were incubated with primary antibodies, such as the anti-PSMD4 antibody (1:200 dilution) in the blocking solution for 1.5 h. Next, the cells were incubated for 40 min with Alexa Fluor 488- or Alexa Flour 594-conjugated secondary antibodies (1:1000 dilution) [18]. The cells were mounted with a DAPI-containing mounting solution (Abcam).

For FRAP analysis, droplet assemblies were bleached in a circular 0.5 µm^2^ region of interest using a 2-s pulse of the 488-nm laser line at full power using a Nikon A1 confocal lase microscope. Recovery was monitored every 2 s for 60 frames. Plotting and curve fitting were performed using Graph Pad Prism 5 (GraphPad Software).

### 2.6. Live-Cell Imaging

For live-cell imaging experiments, the medium was replaced with phenol red-free DMEM (Thermo Fisher Scientific) supplemented with 10% FBS. After the incubation for 12 h, the cells were transferred to an incubator microscope, maintained at 37 °C in 5% CO_2_, and imaged for 12 h.

### 2.7. Terminal Deoxynucleotidyl Transferase dUTP Nick End Labeling (TUNEL) Assay

Apoptosis was determined using the TUNEL Andy Fluor^TM^ 488 Apoptosis Detection Kit (ABPBio) according to the manufacturer’s instructions. Briefly, the cells were washed and fixed as described for immunofluorescence staining. Next, the cells were permeabilized with 0.1% Triton X-100 in 0.1% sodium citrate. The negative control was incubated with Label Solution (without terminal transferase), while the positive control was incubated with recombinant DNase I for 10 min at room temperature to induce DNA strand breaks, prior to labeling procedures. After washing 2 times with PBS, fixed and permeabilized cells were incubated with 50 µL of TUNEL reaction mixture at 37 °C for 60 min in a humid atmosphere in the dark. Finally, cells were directly analyzed under a fluorescence microscope by using an excitation wavelength in the range of 450–500 nm (e.g., 488 nm) and detection in the range of 515–565 nm (green).

### 2.8. Purification of 26S Human Proteasomes

The HEK293 cell line stably expressing biotin-tagged human PSMB2 was used to affinity-purify human proteasome holoenzymes [19,20]. Briefly, after culturing in 15 cm culture dishes, the cells were harvested and lysed with lysis buffer (50 mM NaH_2_PO_4_ (pH 7.5), 100 mM NaCl, 10% glycerol, 5 mM MgCl_2_, 0.5% of NP-40, 5 mM ATP, and 1 mM dithiothreitol, and protease inhibitors). The lysates were then homogenized in a Dounce homogenizer and centrifuged at 16,000× *g* for 15 min at 4 °C. Next, the supernatants were incubated with BioMag Streptavidin resin (Bangs Laboratories) for 6 h at 4 °C. After washing the beads with lysis buffer and TEV buffer (50 mM Tris-HCl, pH 7.5, 1 mM ATP, and 10% glycerol), the 26S proteasome were eluted from the beads by incubating with TEV protease (Invitrogen) in TEV buffer for 1.5 h at 30 °C. The eluted proteasomes were concentrated using Amicon Ultra centrifugal filters.

### 2.9. Size-Exclusion Chromatography

Cell lysates for each condition were prepared by centrifugation twice at 18,000× *g* for 30 min at 4 °C and then loaded onto a Superose 6 Increase 10/300 GL column fast protein liquid chromatography system (ÄKTA, GE Healthcare, Chicago, IL, USA). Elution was carried out using the proteasome SEC buffer (50 mM NaH_2_PO_4_ (pH 7.5), 100 mM NaCl, 5 mM MgCl_2_, 5 mM ATP, and 1 mM DTT). Fractions with volume of 0.2 mL were collected and 10% glycerol was added to each fraction for storage.

## 3. Results

### 3.1. NaCl-Mediated Hyperosmotic Stress Induced the Formation of Nuclear Proteasome Foci

**Figure 1:**
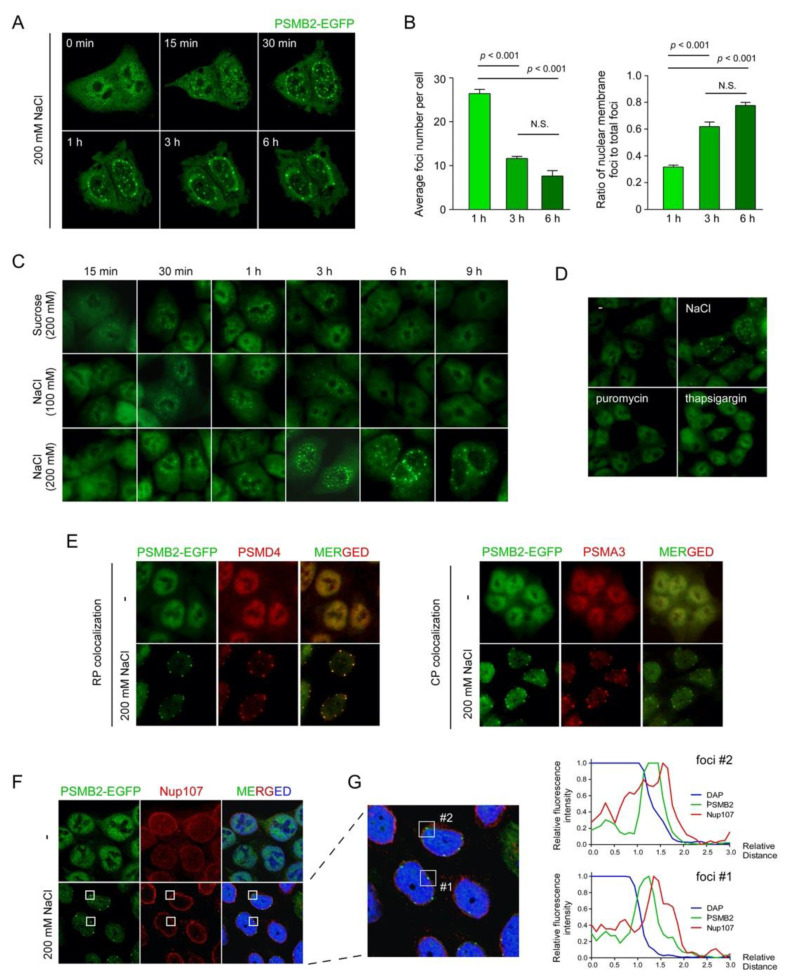
Hyperosmotic stress led to the formation of proteasome foci in the nucleus. (**A**) Time-lapse images of HCT116 cells stably expressing EGFP tagged PSMB2 (HCT116-PSMB2-EGFP cells). The cells were treated with 200 mM NaCl for the indicated time periods. (**B**) Quantification of the number of proteasome foci per cell (*left*) and the number of foci localized near the nuclear membrane (*right*). Cell numbers are 24, 29, and 28, average numbers of total foci (in nucleoplasm and nuclear membrane) per cell are 26.4, 11.6, and 7.6, and average numbers of nuclear membrane foci are 8.4, 7.1, and 5.9, at 1 h, 3 h, and 6 h after NaCl treatment, respectively. Data are analyzed by one-way ANOVA with the Tukey’s multiple comparison tests. (**C**) Cells were treated with 200 mM sucrose, 100 mM NaCl, or 200 mM NaCl for the indicated time periods and the formation of nuclear proteasome foci were examined using fluorescence microscope. (**D**) HCT116-PSMB2-EGFP cells were stimulated by either NaCl (200 mM) for osmotic stress, puromycin (50 µg/mL) for translational inhibition, or thapsigargin (10 µM) for ER stress for 6 h. (**E**) Immunostaining of a proteasome RP subunit PSMD4 (*left*) and CP subunit PSMA3 (*right*). These subunits were colocalized with PSMB2-EGFP signals in the nuclear stress bodies during hyperosmotic stress. (**F**) Immunostaining of nucleoporin 107 (NUP107), a subunit of nuclear pore complex, which was not colocalized with hyperosmotic stress-induced proteasome foci. (**G**) Fluorescence intensities of DAPI, NUP107, and PSMB2 were quantified. Each graph represents the normalized fluorescence distribution in the rectangles containing nuclear proteasome foci.

While investigating the dynamics of nuclear proteasomes, we observed from multiple cell lines that hyperosmotic stress (200 mM NaCl for ~6 h) resulted in not only reduced levels of proteasome subunits in the nuclear fractions, but also spatial changes of nuclear proteasome-positive signals that originally dispersed in the nucleoplasm to become condensed into multiple foci near the anterior nuclear periphery (Figure S1). To monitor the nuclear foci formation of proteasomes, we utilized HCT116-derived knock-in cell lines with an EGFP-tagged version of PSMB2 [15]. EGFP-PSMB2 levels in the knock-in cells appeared to be slightly higher in the nucleus than in the cytosol (Figure 1A). Consistent with the immunostaining results, time-lapse images repeatedly showed the dynamic redistribution of nuclear proteasomes as follows. (1) They started to nucleate as smaller speckles throughout the nucleoplasm after the treatment with 200 mM NaCl for ~1 h. (2) The speckles gradually increased in number and in fluorescence density. (3) The puncta coalesced among themselves and became larger as they moved toward the periphery of the nucleus after ~3 h. (4) The foci were virtually exclusively located and relatively regularly spaced near the nuclear membrane after ~6 h. (5) They became almost immobile at the location without changing their fluorescence intensity by 12 h (Figure 1A and Video S1).

The number of foci gradually decreased with time from 26.4 at 1 h to 7.7 at 6 h (*p* < 0.001) post-hyperosmotic stress, and the ratio of foci contiguous with nuclear membrane to total foci increased from 0.32 to 0.78 by 6 h (*p* < 0.001) (Figure 1B). These observations seem to reflect the phenomenon whereby the proteasome puncta throughout the nucleus merged into bigger foci, simultaneously dissipating from the nucleoplasm to the nuclear membrane. The rapid formation of initial nuclear speckles is thought to be the mechanical response to the osmotic efflux of cellular fluid, rather than complex cellular defense processes.

Other osmolytes such as 200 mM sucrose generated a number of nucleoplasmic foci after ~1 h of treatment (Figure 1C). However, unlike when using the same concentration of NaCl, sucrose-induced PSMB2-EGFP foci were not located near the nuclear membrane and virtually completely disappeared after ~6 h of treatment. Treatment with 100 mM or lower concentrations of sucrose did not result in any puncta formation, but 300 mM sucrose formed similar proteasome foci near the nuclear periphery, as observed upon the NaCl treatment (Figure S2A). Cells treated with 100 mM NaCl had only a limited number of nuclear speckles, but completely lacked the perinuclear foci (Figure S2B). These results indicate that nuclear proteasome foci formation upon hyperosmotic stress is mainly correlated with the severity of hyperosmotic stress and does not differentially respond to diverse osmolytes. Other cellular stresses, such as endoplasmic reticular stress, oxidative stress, translation inhibition, and glucose starvation, did not produce any similar proteasome foci (Figure 1D and data not shown), implying that the observed cellular phenotypes regarding the nuclear proteasome compartmentalization are likely to be limited to the context of hyperosmotic stress.

To examine the possibility that 26S proteasomes were disassembled into the 20S (also known as the core particle or CP) and the 19S (the regulatory particle, RP) complex when redistributed in the nucleus, we immunostained the RP subunit (PSMD4) and the CP subunit (PSMA3) after treating the HCT116-PSMB2-EGFP knock-in cells with 200 mM NaCl for 6 h. The PSMD4- and PSMA3-positive signals strongly overlapped with the PSMB2-EGFP foci contiguous with the nuclear membrane (Figure 1E), strongly suggesting that intact 26S proteasomes were present in the osmostress-induced foci. We consistently observed reduced total cell volume, shrinking nucleolus, and actively forming filopodia during hyperosmotic stress (Figure 1 and data not shown).

We also found that the observed nuclear stress bodies were positioned in the interior of the nuclear envelope (immunostained with lamin A/C) without colocalization with double-stranded DNA (stained with DAPI) (Figure S2C). In addition, immunostaining results, using antibodies targeting the components of nuclear basket components such as nucleoporin 107 (NUP107), revealed that the osmostress-induced proteasome foci were close to but not colocalized in the nuclear pore complex (Figure 1F). They were distributed throughout the space between the condensed chromatin mass and inner nuclear membrane (Figure 1G). Our findings suggest that the proteasome foci are likely to be positioned in a distinct nuclear territory and not directly associated with either chromatin mass or nuclear lamina. A cryo-EM study has identified nuclear proteasomes, as their 26S form, tethered to two specific locations of the nuclear pore complex [21]. Therefore, it seems possible that at least a part of the 26S proteasome is anchored to the basket of nuclear pore complexes under osmotic stress conditions.

### 3.2. The Formation of the Hyperosmotic Stress-Induced Nuclear Proteasome Foci Was Not Mediated by General Osmotic Stress Responses but via Liquid-Liquid Phase Separation

**Figure 2:**
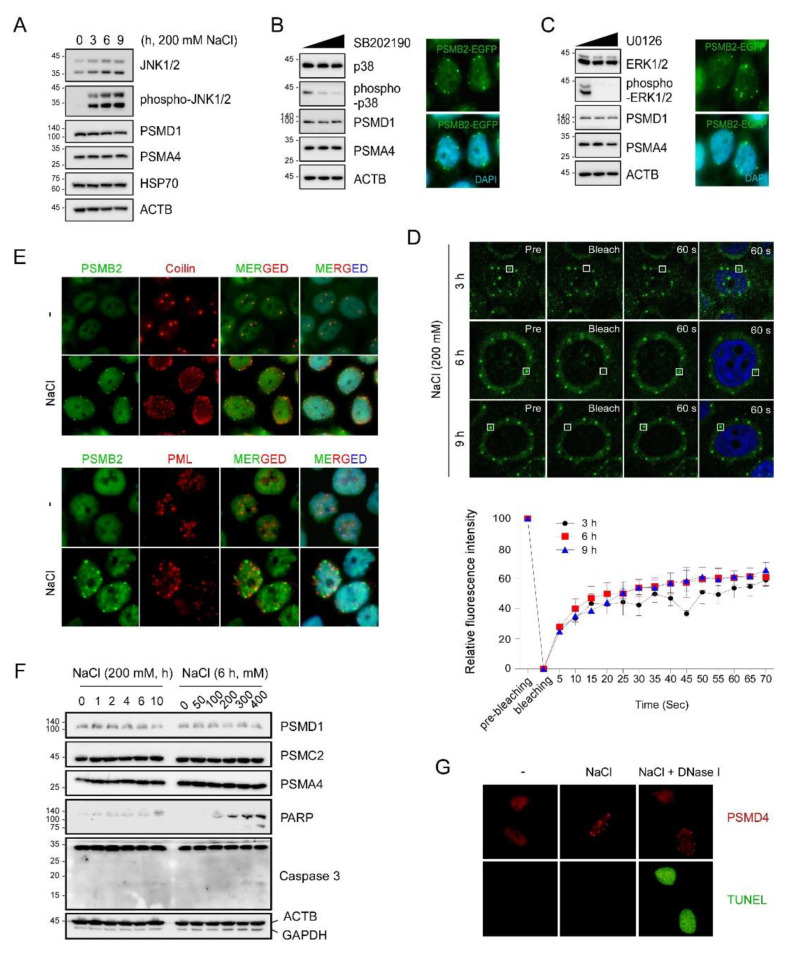
Hyperosmotic stress-induced nuclear puncta of proteasome foci are formed through liquid-liquid phase separation. (**A**) HCT116-PSMB2-EGFP cells were treated with 200 mM NaCl for the indicated time periods and their whole-cell extracts were subjected to SDS-PAGE followed by immunoblotting. (**B**) Cells under hyperosmotic stress were co-treated with 0, 25, and 50 µM of the p38 inhibitor SB202190 for 6 h. The whole cell extracts were subjected to SDS-PAGE/immunoblotting (*left*). Representative images are shown after treatment with 25 µM inhibitors (*right*). (**C**) As in B, except that the ERK inhibitor U0126 was used. (**D**) Top, fluorescence recovery after photobleaching of hyperosmosis (200 mM NaCl for 3, 6, or 9 h)-induced proteasomal foci in the cells. Shown are representative fluorescence images from pre-bleaching (Pre), bleaching, and recovery (60 s) stages on the region of interest (white square). Bottom, quantification of fluorescence recovery. (**E**) HCT116-PSMB2-EGFP cells under hyperosmotic stress were immunostained with coilin (a marker for Cajal bodies) and promyelocytic leukemia proteins (for PML bodies). (**F**) Apoptosis-related proteins were examined by immunoblotting. (**G**) Images of TUNEL-stained cells under hyperosmotic stress (200 mM NaCl for 6 h). For positive control, TUNEL staining was performed using DNAse I.

Cellular adaptive responses under hyperosmotic stress are controlled by many signaling pathways, among which the MAPK signaling pathways, involving extracellular signal-regulated kinase (ERK), p38, and c-Jun N-terminal kinase (JNK), are known to be rapidly activated upon acute hyperosmotic stress to restore cell volume, reorganize the cytoskeleton, and upregulate osmostress-sensing genes [22]. p38 MAPK has been shown to phosphorylate the RP subunit of the 26S proteasome, reduce proteolytic activity, and subsequently elevate the level of global proteasome substrates [11]. Consistent with these previous reports, we observed strong activation of JNK after 200 mM NaCl treatment in the immunoblotting-based analysis (Figure 2A). The levels of proteasome CP and RP subunits in the soluble fractions of whole-cell lysates did not significantly change.

To examine the potential role of p38 in perinuclear proteasome foci formation during osmotic stress, we tested a panel of small-molecule inhibitors. While SB202190 potently blocked p38 activation in response to high osmolarity, there was no effect on proteasome foci, even in the presence of SB202190 (Figure 2B). Furthermore, pharmacological inhibition of other MAPKs, such as MEK, ERK1/2, and JNK, did not prevent the aggregation of nuclear proteasomes during osmostress, and the resulting nuclear bodies had a similar size and morphology, as detected when only osmostress was exerted (Figure 2C and data not shown). The average numbers of nuclear membrane puncta per cell (n = 244, 93, and 106) were 3.64, 3.73, and 3.62 in control, p38-inhibited, and ERK-inhibited cells, respectively. These results imply that the nuclear 26S proteasome condensates formed under hyperosmotic stress may be mainly caused by environmental or mechanical changes, such as water efflux, DNA/protein crowding, and/or elevated ionic strength in the nucleus, rather than mediated by a specific MAPK signaling pathway.

The perinuclear proteasome foci were observed to have a spherical shape, which is a typical property of liquid droplets. Previous studies have also indicated that the nucleoplasmic puncta induced by high concentrations of sucrose exhibited liquid droplet-like properties [15]. We performed fluorescence recovery after the photobleaching (FRAP) assay using the PSMB2-EGFP knock-in cell line. The signal intensity of the perinuclear proteasome foci formed after treatment with 200 mM NaCl was observed to gradually recover after photobeaching (Figure 2D and Videos S2–S4). After 20 min of recovery, more than 50% of the initial fluorescence was recovered. These FRAP assay results clearly indicated that the osmostress-induced nuclear foci were liquid droplets. This cellular response appeared to be independent of the formation of other stress-induced nuclear granules, such as Cajal bodies and promyelocytic leukemia (PML) bodies, which were not observed to be significantly colocalized with the nuclear proteasome foci (Figure 2E).

In addition, there was virtually no overlap between the fluorescence signals from proteasome foci and stress granule marker eIF3E (Figure S3). However, we found that eIF3E-positive signals were significantly elevated in the cytosol after NaCl treatment, suggesting facilitated stress granule assembly in response to hyperosmotic stress. No significant apoptotic markers or apoptotic cells were observed after 6 h of treatment with 200 mM NaCl (Figure 2F,G). Proteasome foci formation may be cytoprotective under hyperosmotic stress conditions that otherwise trigger cell death. As recently identified, they may function as a key nucleoplasmic proteolytic center, actively eliminating the apoptosis-inducing orphan ribosomal proteins [15,23,24]. The number, size, and unique nuclear distribution of proteasome foci are distinguishable from the recently identified p62 nuclear bodies [24], but highly resemble those described for chromatin insulator bodies, which are also formed in response to hyperosmolarity and were reported to localize to the nuclear periphery with chromatin-associated proteins [16]. Taken together, our data indicate that the proteasome foci formed under hyperosmotic stress conditions are likely phase separation-induced liquid droplets and are functionally important for stress response.

### 3.3. Formation of Nuclear Proteasome Foci Was Affected by Proteasome Transport between the Nucleus and Cytosol

**Figure 3:**
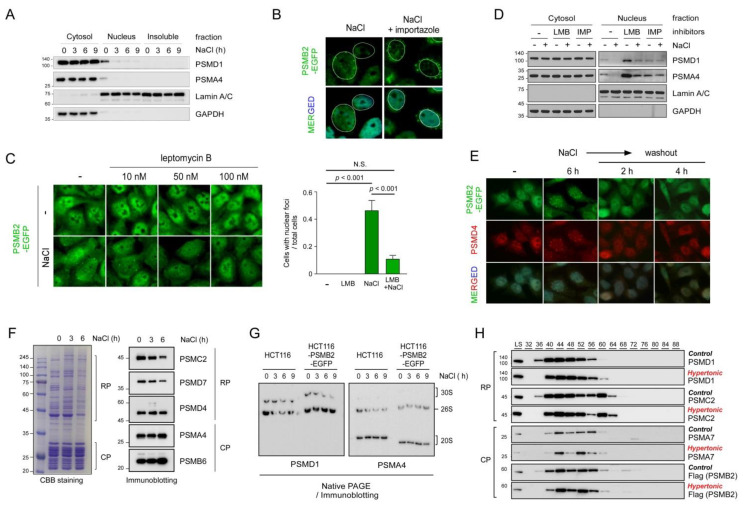
The nucleocytoplasmic transport of 26S proteasomes is implicated in the nuclear proteasome foci formed during hyperosmotic stress. (**A**) Immunoblotting analysis of a CP subunit (PSMA4) and a RP subunit (PSMD1) was conducted using the cytosolic and nuclear fractions of whole-cell lysates from hyperosmosis-stressed HCT116-PSMB2-EGFP cells. (**B**) Cells were treated with 200 mM NaCl in the absence or presence of 40 µM importazole (a nuclear import inhibitor) for 6 h. White circles indicate the nuclear membrane. (**C**) As in B, except that the cells were treated with the indicated concentrations of leptomycin B (a nuclear export inhibitor). Data are analyzed by one-way ANOVA with the Tukey’s multiple comparison tests. (**D**) As in A, except that the cells were cotreated with importazole (40 µM) or leptomycin B (50 nM). (**E**) During washing out NaCl with a normal medium, changes in PSMB2-EGFP and PSMD4-positive immunostaining signals were monitored. (**F**) Human 26S proteasomes were affinity-purified from HEK293 cells stably overexpressed PSMB2-biotin before and after treatment with 200 mM NaCl. The purified proteasomes analyzed by SDS-PAGE followed by Coomassie brilliant blue (CBB) staining and immunoblotting. (**G**) The integrity and level of proteasomes were examined using non-denaturing (native) PAGE and the whole-cell lysates from HCT116 cells and HCT116-PSMB2-EGFP cells. (**H**) Size-exclusion chromatography of whole-cell extracts from HCT116-PSMB2-EGFP cells treated with NaCl (200 mM, 6 h). Components of 26S proteasomes were examined by SDS-PAGE/immunoblotting.

We next fractionated the NP-40-solublized whole-cell extracts and examined the 26S proteasome in the cytosol and nucleus. While the number of proteasomes in the cytosolic fraction appeared to be comparable before and after the hypertonic stress condition, in a stark contrast, a significant amount of the nuclear proteasomes disappeared in a time-dependent manner after the treatment with 200 mM NaCl (Figure 3A). On the contrary, no proteasome subunits were detected in the insoluble fraction after NaCl treatment, suggesting that the nuclear foci, although they were generated from LLPS, may still have more soluble-like characteristics. To gain insight into the dynamics of the nuclear proteasome, we subsequently tested whether importazole, which inhibits importin-β−mediated nuclear import [25], affected the formation of hyperosmotic stress-induced nuclear proteasome foci. Rather than forming the nuclear puncta which had a similar morphology to the nuclear insulator body, importazole-treated cells completely failed to generate intranuclear foci, but instead exhibited cytosolic foci at the exterior of the perinuclear region (Figure 3B). We then blocked the nuclear export process using leptomycin B, a specific inhibitor of exportin 1, during the osmotic stress response. In contrast to the effects of importazole, treatment with leptomycin B effectively blocked the formation of proteasome foci both in the cytosol and nucleus. PSMB2-EGFP signals remained diffusely distributed in the nucleus, as observed under stress-free conditions (Figure 3C).

Consistent with the microscopic data, the levels of proteasome subunits were found to have drastically increased in the nuclear fraction, but not in the cytosolic fraction, under the hyperosmotic stress condition (Figure 3D). However, treatment with leptomycin B effectively abrogated this phenomenon. We did not observe any significant changes in cytosolic proteasome amounts under the same conditions, likely due to the excess level of the proteasome in the cytosol and the more soluble biophysical characteristics of the cytosolic proteasome stress granules. Overall, our results strongly suggest that nuclear export/import machinery gated by the nuclear pore complex is required for the formation of nuclear proteasome foci during hyperosmotic stress. Notably, both importins and exportins, which are critical regulators of nucleocytoplasmic transport, have been previously identified as the components of stress granules [26,27].

To examine whether the osmotic stress-induced nuclear proteasome foci formed in a reversible manner, NaCl-treated cells were returned to normal (isotonic) medium. We found that the puncta mostly disappeared in less than 30 min after the removal of NaCl as the cell morphology returned to the stress-free state (Figure 3E). The numbers of cells with nuclear membrane foci were 88 (77.9%), 7 (9.3%), and 4 (3.4%), out of 113, 75, and 116 total cells at 0 h, 2 h, and 4 h post-washout, respectively. Both CP and RP subunits (PSMB2 and PSMD4, respectively) showed similar behaviors in the washout experiments (Figure 3E). The dynamic changes in 26S proteasomes during hyperosmotic stress were biochemically dissected using purified proteasomes from the HEK293-derived cell line overexpressing PSMB2-biotin. The overall integrity and abundance of 26S proteasomes under normal and osmotic stress conditions were comparable (Figure 3F). After 6 h of treatment with 200 mM NaCl, 26S proteasomes in the whole-cell lysates also showed no significant changes in the levels of CP or RP complexes (Figure 3G). The biochemical changes in cellular proteasomes were further investigated using size-exclusion chromatography, where CP and RP subunits were largely eluted in the same fraction, regardless of the osmotic stress (Figure 3H). These data indicate little structural reorganization of nuclear proteasomes without CP-RP disassociation under acute hyperosmotic stress, although further detailed biochemical analysis is required.

### 3.4. Stress Granules and Damaged Nuclear Pore Complexes Were Linked to Nuclear Proteasome Foci under Hyperosmotic Stress

We found that co-treatment with the proteasome inhibitor MG132 along with hyperosmotic stress resulted in a completely different distribution of cellular proteasomes from when only osmostress was exerted. It was observed to lead to the extensive formation of both nuclear and cytosolic proteasome puncta (Figure S4A). Washing out the cells with normal (isotonic and without MG132) media quickly diffused the proteasome-positive speckles, indicating the highly dynamic nature of the proteasome foci throughout the cells. NaCl-induced proteasome puncta in the nucleus were colocalized with K48-linked ubiquitin chains (Figure S4B), consistent with previous reports [15,28]. However, the notion that the nuclear proteasome bodies function as an active proteolytic center for polyubiquitin conjugates needs to be validated because osmostress is usually accompanied by ATP deficiency. In contrast, nuclear foci formation was completely inhibited by pre-treatment with MLN-7243, an inhibitor of the ubiquitin-activating enzyme E1 (Figure S4C), suggesting the coalescence of non-degraded polyubiquitin conjugates as key condensed biomolecules in liquid droplets and, consequently, foci formation.

Stress granules, known to have liquid droplet-like properties and cytoprotective roles, are major stress-responsive, membrane-less compartments in the cytoplasm that are usually generated through defective cellular translation processes caused by a wide array of stress [9]. Based on the hypothesis that stress granules may be involved in the nuclear foci formation, we first immunostained the stress granule core proteins, such as G3BP and PABP, and found that their signals as cytoplasmic puncta were significantly increased upon the hyperosmotic stress (Figure S4D). The morphology, size, and number of stress granules induced by osmostress were identical to those triggered by MG132, arsenite, or heat shock [29,30,31]. In addition, we observed that some nuclear pore complex signals (immunostained with NUP358 in HCT116 cells) were detectable in the cytoplasm and were highly colocalized with stress granule markers (Figure S4D). Stress granule assembly has been reported to negatively regulate nucleocytoplasmic transport through the spatial rearrangement of nuclear pore complex components [27]. Therefore, these results have raised a possibility that hypertonic stress disrupts nucleocytoplasmic transport by recruiting transport components from the nuclear membrane to stress granules. The nuclear proteasome foci may function as degradation sites for misfolded proteins and ribosomal proteins, possibly in an ATP- and ubiquitin-independent manner.

## 4. Discussion

**Figure 4:**
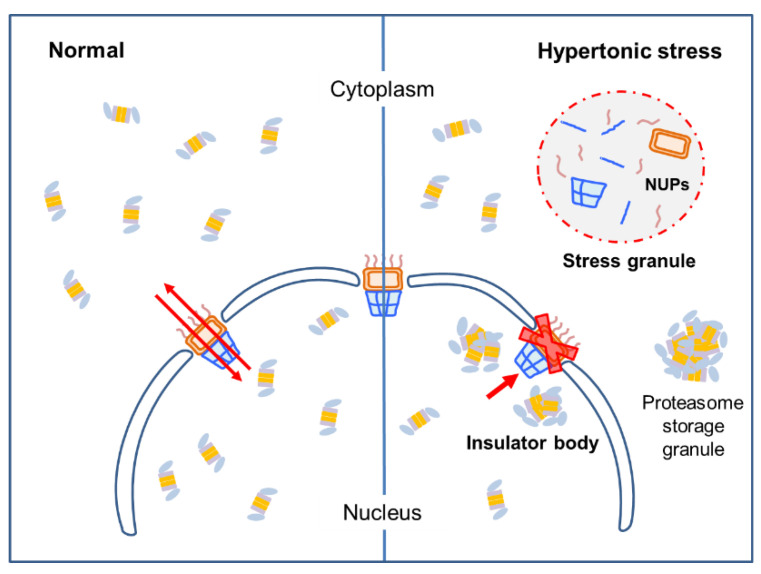
Proposed model for nuclear proteasome foci formation. Unstressed cells freely translocate 26S proteasomes from the nucleus to cytosol and vice versa through NPCs. However, cells under hyperosmotic environments appear to lose this normal transport function potentially through the sequestration of NPC components into stress granules. Both nucleoplasmic proteasome foci and cytoplasmic stress granules are membraneless organelles formed through LLPS or demixing among intact 26S proteasome holoenzymes and their substrates. Whether hyperosmotic stress leads to the formation of cytoplasmic proteasome body, such as proteasome storage granules, remains to be identified.

Based on our findings, we propose that the impaired nucleocytoplasmic transport of nuclear proteasomes may contribute to the formation of peri-nucleic proteasome foci along with their spontaneous assembly through LLPS in the nucleus during cellular hypertonic stress responses (Figure 4). Consistent with this hypothesis, they exhibited dynamic and liquid droplet-like characteristics, which may rapidly (without complex signaling cascades) sequestrate the key proteins in the nucleus under hyperosmotic stress condition. In addition, unlike insoluble aggregates, they could quickly and reversibly restore their normal function when stress is relieved. The contribution of increased nuclear protein concentration (by water efflux under cellular hyperosmotic stress conditions) on the phase separation mechanism has not yet been clearly characterized. However, our study indicates that not only specific signaling pathways, such as the p38 MAPK pathway, but also general mechanistic forces can contribute to cellular stress response strategies. It is likely that the observed phenomenon here is evolutionarily conserved. Both hyperosmotic stress and nucleocytoplasmic transport defects are involved in critical pathological events in many human diseases. Further understanding of this pathway will provide effective therapeutic strategies to pharmacologically modulate the stress-related cellular responses.

## Supplementary Materials

The following are available online at https://www.mdpi.com/article/10.3390/cells10092493/s1, Figure S1: Hypertonic solution induced nuclear proteasome foci formation in multiple cell lines., Figure S2: The cellular response upon hyperosmotic stress was dependent on osmolyte concentration, Figure S3: Formation of nuclear proteasome foci were linked with stress granules, Figure S4: The ubiquitin-proteasome system is involved in the formation of nuclear proteasome foci, Video S1: Formation of nuclear proteasome foci when cells are treated with 200 mM NaCl, Video S2–S4: FRAP analysis performed after cells were treated with 200 mM NaCl for 3 h (S2), 6 h (S3), and 9 h (S4).
