# Supplementary material for: Formation of Non-Nucleoplasmic Proteasome Foci during the Late Stage of Hyperosmotic Stress
Source: Cells. 2021 Sep 21;10(9):2493. doi: 10.3390/cells10092493 (PMC8467775; doi:10.3390/cells10092493)
Supplement: Supplementary file 1 [file cells-10-02493-s001.zip › cells-1330030-supple.pdf]

SUPPLEMENTARY FIGURES

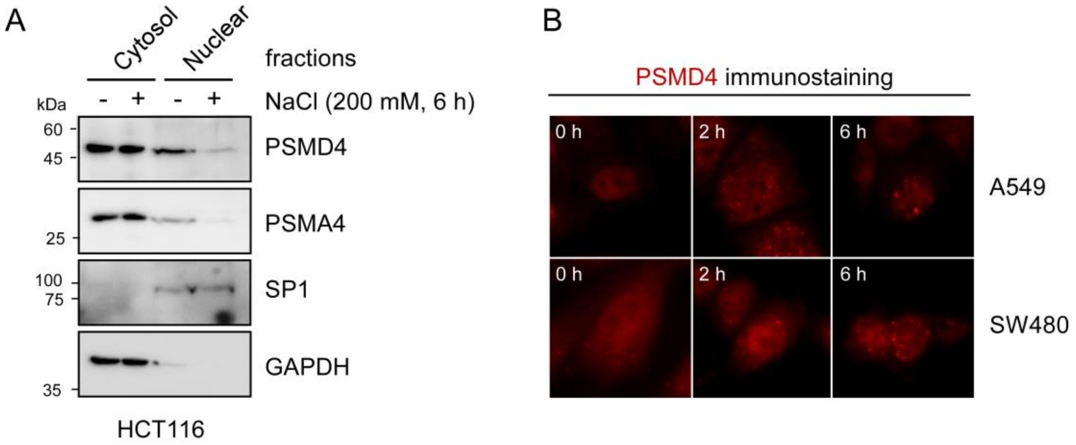

Figure S1. Hypertonic solution induced nuclear proteasome foci formation in multiple cell lines. (A) Whole-cell lysates of HCT116 cells before and after treated with 200 mM NaCl for 6 h were separated to the cytosolic and nuclear fractions, which were subjected to SDS-PAGE/immunoblotting. The transcription factor specificity protein 1 (SP1) and glyceraldehyde-3-phosphate dehydrogenase (GAPDH) were used as markers for nucleic and cytoplasmic fractions, respectively, after fractionation. These data supplement Figure 3A. (B) The lung cancer cell A549 and the colon cancer cell SW480 were treated with 200 mM NaCl for the indicated times and analyzed with immunostaining with anti-PSMD4 antibodies (red).

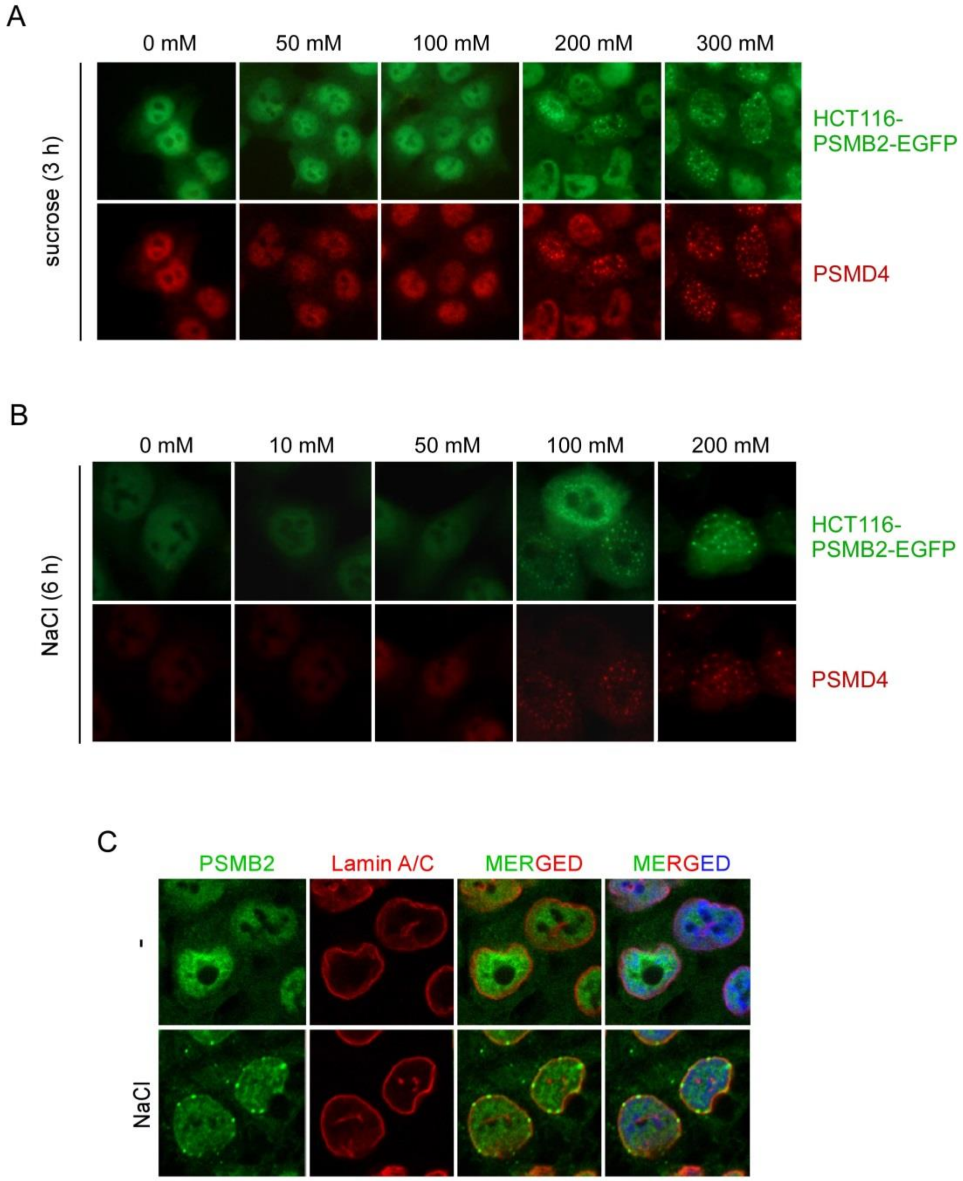

Figure S2. **The cellular response upon hyperosmotic stress was dependent on osmolyte concentration.** (A) HCT116-PSMB2-EGFP cells were treated with the indicated concentrations of NaCl for 6 h and PSMB2-EGFP fluorescence along with PSMD4 immunostaining-positive signals were monitored. (B) As in A, except that sucrose was used for hypertonic stress for 3 h. (C) Cells were immunostained for lamin A/C, a nuclear membrane marker.

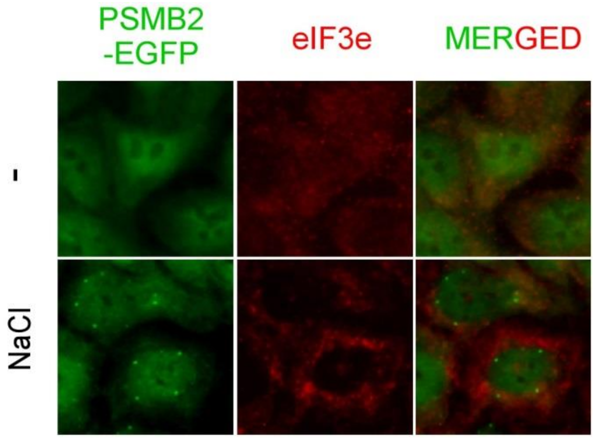

Figure S3. Formation of nuclear proteasome foci were linked with stress granules. HCT116-PSMB2-EGFP cells under osmotic stress (200 mM NaCl for 6 h) were immunostained with anti-eIF3e antibodies.

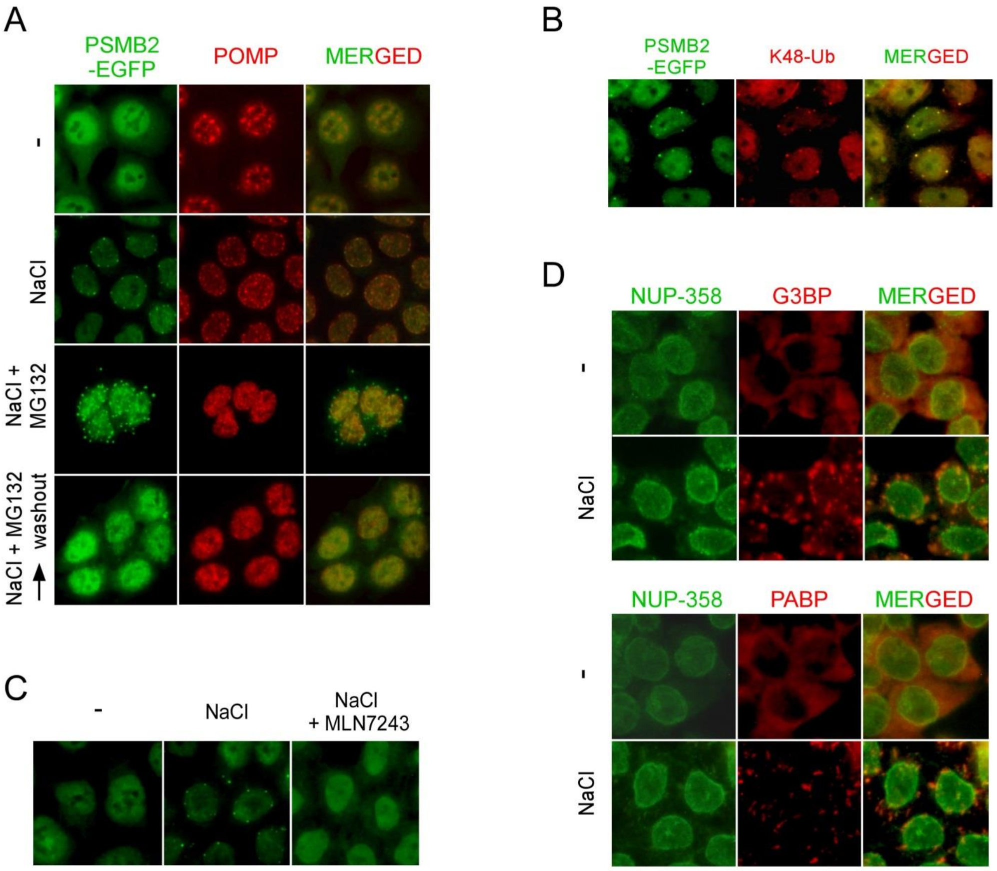

Supplementary Figure 4. **Nuclear proteasome foci under hyperosmotic stress were potentially linked with stress granules and defects in the nuclear pore complexes.** (A) HCT116-PSMB2-EGFP cells were treated 200 mM NaCl for 6 h in the absence and presence of 10 μM MG132, washed-out for 2 h, and then immunostained with the proteasome maturation protein POMP. (B) HCT116-PSMB2-EGFP cells were treated with 200 mM NaCl for 6 h and endogenous Lys48-linked polyubiquitin chains (K48-Ub) were immunostained. (C) Cells were stimulated with NaCl (200 mM, 6 h) in a absence or presence of the ubiquitin activating (E1) enzyme inhibitor MLN-7243. (D) Cells under hyperosmotic stress were co-immunostained with NUP358, a subunit of the nuclear pore complex, and stress granule markers such as G3BP and PABP in the cytoplasm.

SUPPLEMENTARY MOVIES

Video S1. Formation of nuclear proteasome foci when cells are treated with 200 mM NaCl
PSMB2-EGFP fluorescence time-lapse movie after cells were treated with 200 mM NaCl.

Video S2-S4. FRAP analysis performed after cells were treated with 200 mM NaCl
FRAP analysis on nuclear proteasome foci formed after 3 h (Video S2), 6 h (Video S3), and 9 h (Video S4) of 200 mM NaCl treatment.
